# Accelerating spiral tissue phase velocity mapping without affecting peak velocity measurements

**DOI:** 10.1186/1532-429X-16-S1-W31

**Published:** 2014-01-16

**Authors:** Robin Simpson, Jennifer Keegan, Peter Gatehouse, Michael Hansen, David Firmin

**Affiliations:** 1 Royal Brompton Hospital, Imperial College, London, UK; 2 National Heart, Lung and Blood Institute, NIH, Bethesda, Maryland, USA

## Background

TPVM is a promising technique for measuring myocardial mechanics [1] but long scan times have limited its clinical application, despite acceleration techniques such as view-sharing [2] or k-t BLAST [3] being used. Cartesian work using k-t BLAST found that accelerations greater than a factor of 2 affected peak velocity measurements, possibly because of temporal smoothing inherent in the technique [3]. Recently spiral trajectories have been shown to be capable of greatly reducing scan duration in comparison with similar resolution Cartesian sequences [4]. A new spiral TPVM sequence which uses the Gadgetron [5] GPU implementation of non Cartesian SENSE has been developed: this abstract compares peak velocities measured with different acceleration factors.

## Methods

K-space is fully sampled with 8 spiral interleaves (14 ms duration, TR 24 ms). Data is acquired at 2 levels of acceleration, acquiring 4 (R2) and 3 (R3) out of the 8 spirals in order to assess the effect of acceleration on the measured velocities. Velocity compensated and encoded data are acquired in consecutive heartbeats, with an initial heartbeat used to collect coil sensitivity information (breath-hold durations are 17 and 13 heartbeats for R2 and R3). Acquired spatial resolution is 1.7 × 1.7 mm (reconstructed pixel size 0.85 × 0.85 mm). Retrospective cardiac gating is used to cover the entire cardiac cycle (50 phases reconstructed). Images are passed to the Gadgetron for reconstruction and returned to the scanner for viewing within 1 min 20 s. Basal, mid and apical short-axis slices were acquired in 10 healthy volunteers on a Siemens Skyra 3T scanner.

## Results

**Figure 1:**
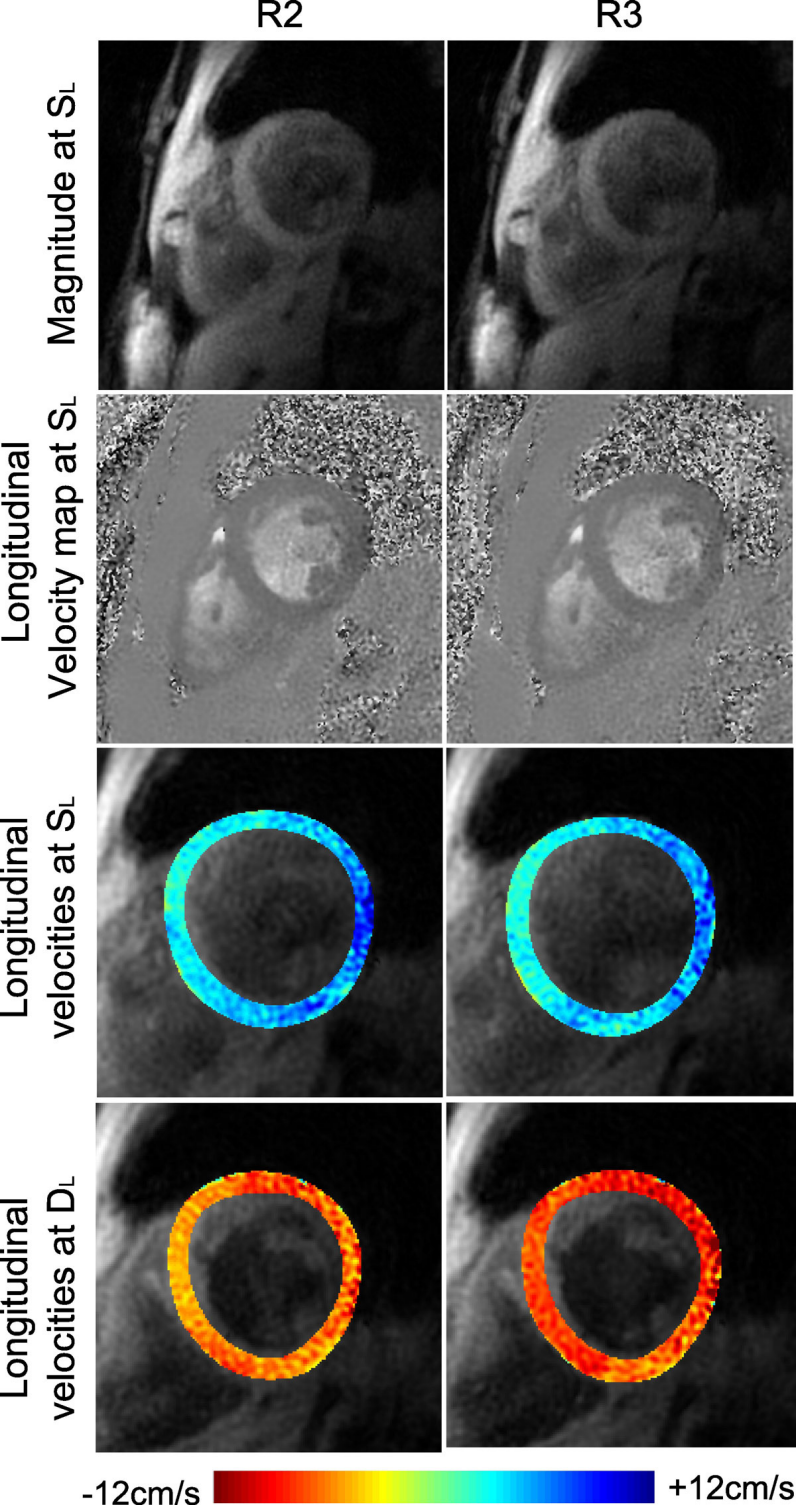
**Magnitude images (top) and longitudinal velocity maps (2nd row), along with colour overlays showing peak systolic (S_L_, 3rd row) and peak diastolic (D_L_, bottom) velocities measured with R2 (left) and R3 (right)**. Image quality is very similar between the two levels of acceleration and measured velocities are qualitatively also very similar.

**Figure 2:**
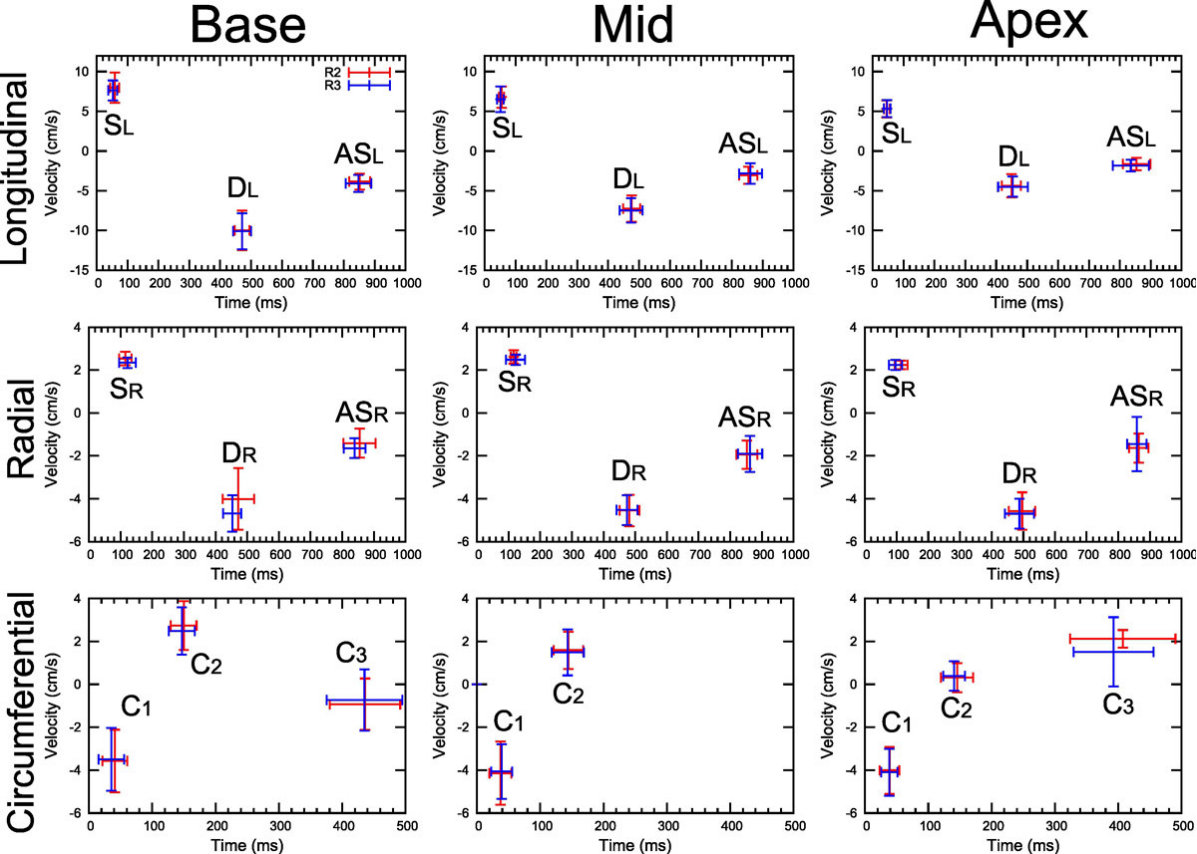
**R2 (red) and R3 (blue) peak and global TTP values shown as mean +/- SD values over the ten volunteers**. A systolic longitudinal (S_L_) and radial (S_R_) peak is seen as well as an early diastolic (D_L _and D_R_) and atrial systolic (AS_L _and AS_R_) peak in each slice. Two systolic circumferential peaks are seen in each slice (C_1 _and C_2_) and a third peak is seen at base and apex in early diastole (C_3_). R2 and R3 show very similar values, indicating that the level of acceleration is not affecting velocity measurements.

Example images and velocity maps for R2 and R3 are shown in Figure 1. Very little difference in image quality or measured velocities is seen between levels of acceleration. Figure 2 shows the mean and SD peak velocity values for R2 and R3 in all three slices and all three directions. Velocities measured with R2 and R3 were extremely similar, with no significant differences between measured peaks or TTPs.

## Conclusions

The use of spiral trajectories, non-Cartesian SENSE and the Gadgetron GPU reconstruction framework has allowed the acquisition of high temporal resolution TPVM images within a breath-hold time that is easily achievable in the clinical environment (13 heartbeats), while reconstruction time is short enough to allow viewing at the time of scanning. The acceleration is not affecting peak velocity measurements (comparisons with previous unaccelerated spiral data [3] also suggest this).

## Funding

NHIR CBRU, Royal Brompton Hospital. HRUK grant RG2584.
